# Differential Effects of Ruminant and Industrial 18-Carbon *trans*-Monounsaturated Fatty Acids (*trans* Vaccenic and Elaidic) on the Inflammatory Responses of an Endothelial Cell Line

**DOI:** 10.3390/molecules26195834

**Published:** 2021-09-26

**Authors:** Carina A. Valenzuela, Ella J. Baker, Camila O. De Souza, Elizabeth A. Miles, Philip C. Calder

**Affiliations:** 1 School of Human Development and Health, Faculty of Medicine, University of Southampton, Southampton SO16 6YD, UK; E.Baker@soton.ac.uk (E.J.B.); Camila.oliveiradesouza@utsouthwestern.edu (C.O.D.S.); e.a.miles@soton.ac.uk (E.A.M.); pcc@soton.ac.uk (P.C.C.); 2 School of Nutrition, Faculty of Pharmacy, University of Valparaíso, Playa Ancha, Valparaíso 2360102, Chile; 3 Department of Cell and Developmental Biology, Institute of Biomedical Sciences, University of São Paulo, São Paulo CEP 05508-000, SP, Brazil; 4 NIHR Southampton Biomedical Research Centre, University Hospital Southampton NHS Foundation Trust and University of Southampton, Southampton SO16 6YD, UK

**Keywords:** *trans* fatty acids, elaidic acid, *trans* vaccenic acid, inflammation, atherosclerosis

## Abstract

Endothelial dysfunction and inflammation are recognised factors in the development of atherosclerosis. Evidence suggests that intake of industrial *trans* fatty acids (TFAs) promotes endothelial dysfunction, while ruminant TFAs may have the opposite effect. The aim of this study was to compare the effects of elaidic acid (EA (18:1n-9t); an industrially produced TFA) and *trans* vaccenic acid (TVA (18:1n-7t); a natural TFA found in ruminant milk and meat) on inflammatory responses of endothelial cells (ECs). ECs (EA.hy926 cells) were cultured under standard conditions and exposed to TFAs (1 to 50 μM) for 48 h. Then, the cells were cultured for a further 6 or 24 h with tumour necrosis factor alpha (TNF-α, 1 ng/mL) as an inflammatory stimulant. ECs remained viable after treatments. TFAs were incorporated into ECs in a dose-dependent manner. Preincubation with EA (50 µM) increased production of MCP-1, RANTES, and IL-8 in response to TNF-α, while preincubation with TVA (1 µM) decreased production of ICAM-1 and RANTES in response to TNF-α. Preincubation with EA (50 µM) upregulated toll-like receptor 4 and cyclooxygenase 2 gene expression in response to TNF-α. In contrast, preincubation with TVA (1 µM) downregulated TNF-α induced nuclear factor kappa B subunit 1 gene expression. Preincubation of ECs with EA (50 µM) increased THP-1 monocyte adhesion. In contrast, preincubation of ECs with TVA (1 µM) reduced THP-1 monocyte adhesion, while preincubation of ECs with TVA (50 µM) decreased the level of surface expression of ICAM-1 seen following TNF-α stimulation. The results suggest that TVA has some anti-inflammatory properties, while EA enhances the response to an inflammatory stimulus. These findings suggest differential effects induced by the TFAs tested, fitting with the idea that industrial TFAs and ruminant TFAs can have different and perhaps opposing biological actions in an inflammatory context.

## 1. Introduction

Coronary heart disease (CHD) and stroke are still the leading causes of death globally [1], and atherosclerosis has a key role in their pathogenesis. Atherosclerosis is a lipoprotein-driven disease that leads to plaque formation at focal areas in the arterial blood vessels through intimal inflammation, necrosis, fibrosis, and calcification [2]. Low-grade chronic inflammation is a feature of obesity and has been related to insulin resistance and atherosclerosis [3,4]. Endothelial dysfunction is a pro-inflammatory state characterised by chronic activation of the endothelium, which leads to atherosclerosis and cardiovascular disease (CVD). The vascular endothelium plays a key role in maintaining vascular homeostasis by regulating vascular tone and permeability, thrombosis, haemostasis, and other inflammatory responses [5,6].

High intake of *trans* fatty acids (TFAs) is associated with an increased risk of CVD [7,8,9,10], which explains the recommendation that these fatty acids (FAs) should contribute <1% of daily energy intake [11]. However, there are two different dietary sources of TFAs: the industrially produced TFAs (iTFAs) and the naturally occurring or ruminant TFAs (rTFAs). iTFAs are produced industrially by partial hydrogenation of vegetable oils and are found in ultra-processed or ready-to-eat foods such as fried foods, fast foods, pastries, shortenings, cake mixes, and many frozen meals and packaged foods [12]. In contrast, rTFAs are generated by biohydrogenation of unsaturated FAs in the rumen of grass grazing sheep and cattle and other ruminants by bacterial isomerases, and therefore they are found in the milk, dairy products, and meat of these ruminant animals [12]. Given that partially hydrogenated vegetable oils can contain between 30 and 50% of iTFAs, mainly elaidic acid (EA; 18:1n-9t), while dairy and ruminant products only have between 2 and 6% of rTFAs (mostly *trans* vaccenic acid, (TVA; 18:1n-7t)), quantitatively the main dietary source of TFAs is usually partially hydrogenated vegetable oils [13].

The evidence from observational studies suggests that higher CVD risk is related to consumption of iTFAs rather than rTFAs, which can be explained, at least in part, by their differential effects on lipoproteins such as low-density lipoprotein (LDL) and high-density lipoprotein (HDL) cholesterol, as well as inflammatory mechanisms [14,15,16]. However, it is not clear how specific TFA isomers differ in their biological activity and mechanisms of action with regard to inflammation.

There are not many studies comparing the effects of TFAs of industrial and ruminant origin in models of endothelial inflammation. In vitro studies, both under basal conditions and after exposure to inflammatory stimuli, usually show that iTFAs have pro-inflammatory effects [17,18,19], while rTFAs have null or the opposite effects to iTFAs [17,20], although the evidence is not always consistent [21,22]. It is difficult to compare the studies testing the effects of TFAs of different origins on inflammatory processes in endothelial cells (ECs), and in other cells, given the methodological differences between them, particularly the use of high concentrations of these TFAs, which may be considered unphysiological when compared with TFA levels reported in human blood [13].

In view of the need for greater knowledge regarding the possible differential effects of TFAs on inflammation, the aim of this study was to compare the inflammatory responses in cultured EA.hy926 ECs exposed to the most common 18-carbon TFAs of both industrial and ruminant origin (EA and TVA) and their *cis* isomers oleic (OA; 18:1n-9) and *cis* vaccenic acid (CVA; 18:1n-7), after stimulation with the inflammatory cytokine tumour necrosis factor (TNF-α). The objective of this study was to gain insight into the mechanisms through which TFAs in general, and 18-carbon iTFAs and rTFAs in particular, affect inflammation and the functioning of human ECs, which is important in relation to development of atherosclerosis and the consequent risk for CVD.

## 2. Results

### 2.1. Viability of EA.hy926 Cells Incubated with TNF-α and FAs

**Figure 1:**
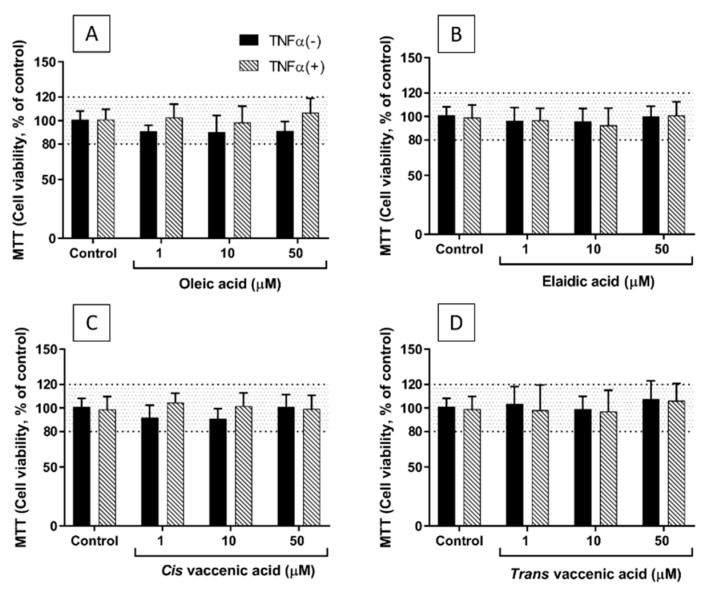
Viability of EA.hy926 cells after preincubation for 48 h with supplemented DMEM containing 0.1% of ethanol (Control) or different concentrations (1, 10, and 50 µM) of oleic acid (**A**), elaidic acid (**B**), *cis* vaccenic acid (**C**), or *trans* vaccenic acid (**D**), followed by incubation with (+) or without (−) TNF-α (1 ng/mL) for 24 h. Bars are mean ± SEM of 9 samples performed in 3 experiments. Data were analysed using two-way ANOVA with Tukey’s post hoc test. There were no significant effects observed.

Neither TNF-α (1 ng/mL for 24 h) nor any of the four FAs tested at concentrations of 1, 10, and 50 µM affected the viability of EA.hy926 cells, as assessed using the 3-(4,5-dimethylthiazol-2-yl)-2,5-diphenyltetrazolium bromide (MTT) assay (Figure 1). However, at a concentration of 100 µM, both EA and TVA reduced viability by an average of 20%. Therefore, further experiments did not use FAs at concentrations above 50 µM. The effects of OA and CVA on EC viability at a concentration of 100 µM were not tested.

### 2.2. FA Incorporation into EA.hy926 Cells

**Figure 2:**
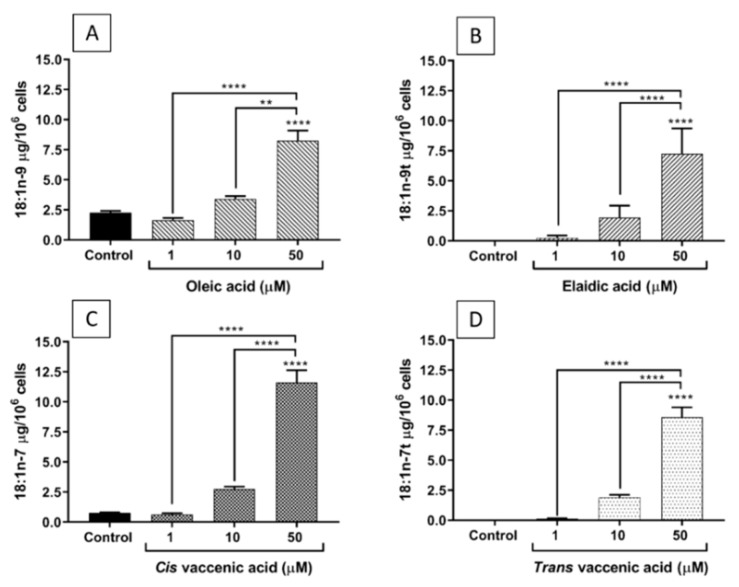
Incorporation of FAs into EA.hy926 cells incubated for 48 h with DMEM containing 0.1% of ethanol (Control) or different concentrations (1, 10, and 50 µM) of oleic acid (**A**), elaidic acid (**B**), *cis* vaccenic acid (**C**), or *trans* vaccenic acid (**D**). Bars are mean ± SEM of 6 to 9 samples performed in 3 experiments. Data were analysed using one-way ANOVA with Tukey’s post hoc test. ** *p* < 0.01; **** *p* < 0.0001 between groups indicated by joined lines or vs. Control when there is no joined line.

Figure 2 shows that the incorporation of each of the studied FAs into EA.hy926 cells increased as their concentration in the culture medium increased from 1 to 50 µM. CVA was incorporated in a higher amount than TVA, while OA and EA were incorporated to a similar extent.

### 2.3. Effects of FAs on the Levels of Inflammatory Mediators Produced by ECs

**Figure 3:**
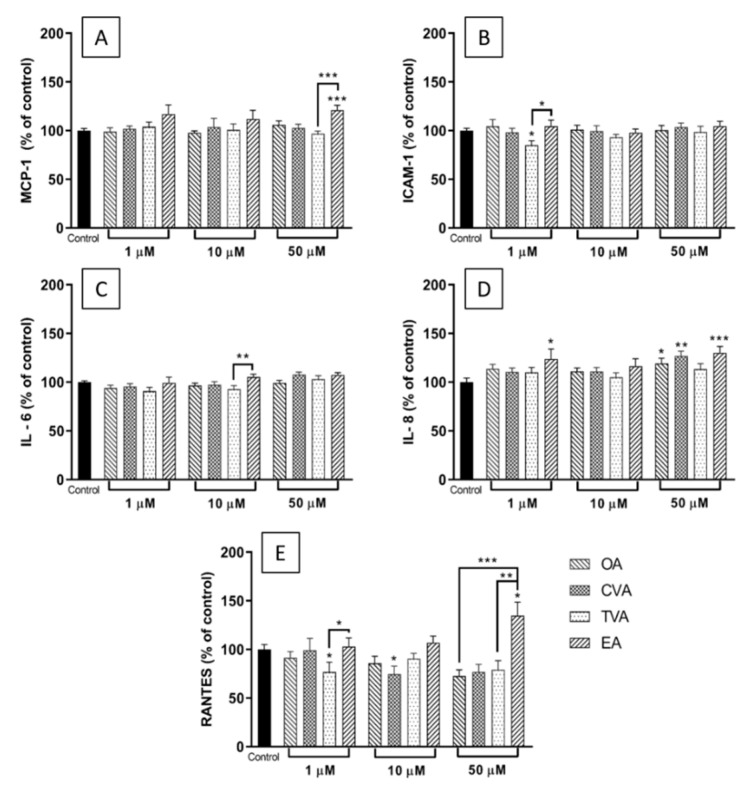
Concentrations (% of control) of MCP-1 (**A**), ICAM-1 (**B**), IL-6 (**C**), IL-8 (**D**), and RANTES (**E**) in the medium of EA.hy926 cells preincubated for 48 h with DMEM containing 0.1% of ethanol (Control) or FA at 1, 20, or 50 µM, followed by incubation with TNF-α (1 ng/mL) for 24 h. Bars are mean ± SEM of 9 samples from 3 experiments. Data were analysed using one-way ANOVA with Tukey’s post hoc test. * *p* < 0.05; ** *p* < 0.01; *** *p* < 0.001 between groups indicated by joined lines or vs. Control when there is no joined line. OA = oleic acid, CVA = *cis* vaccenic acid, TVA = *trans* vaccenic acid, EA = elaidic acid.

Figure 3A shows that preincubation of EA.hy926 cells with any of the four FAs at a concentration of 1 or 10 µM prior to TNF-α stimulation did not induce changes in the supernatant levels of monocyte chemoattractant protein (MCP)-1 compared to control (i.e., TNF-α without any FA preincubation). However, preincubation with 50 µM EA prior to TNF-α stimulation produced a significant increase of MCP-1 levels, compared to control and to TVA pretreated cells (both *p* < 0.001).

Preincubation with TVA at 1 µM produced significant decreases in the supernatant levels of intercellular adhesion molecule (ICAM)-1 in response to TNF-α, compared to control and to EA pretreated cells (both *p* < 0.05) (Figure 3B).

FA preincubation did not induce significant changes in TNF-α induced interleukin (IL)-6 levels compared to control, although preincubation with EA at 10 µM prior to TNF-α stimulation resulted in higher IL-6 levels than preincubation with TVA (*p* < 0.01) (Figure 3C).

Preincubation with EA at 1 or 50 µM before TNF-α stimulation produced a significant increase in IL-8 levels in the supernatant compared with control (*p* < 0.05 and *p* < 0.001, respectively), which was also the case for OA and CVA at 50 µM (*p* < 0.05 and *p* < 0.01, respectively) (Figure 3D).

For regulated upon activation, normal T cell expressed and presumably secreted (RANTES), preincubation with CVA or TVA induced significant changes compared with control, with decreased levels compared to control for CVA at 10 µM (*p* < 0.05) and for TVA at 1 µM (*p* < 0.05). Additionally, preincubation with 50 µM EA significantly increased levels of RANTES compared to control (*p* < 0.05), and compared to preincubation with OA or TVA (*p* < 0.001 and *p* < 0.01, respectively) (Figure 3E).

### 2.4. Effects of FAs on the Expression of Inflammation-Related Genes

**Figure 4:**
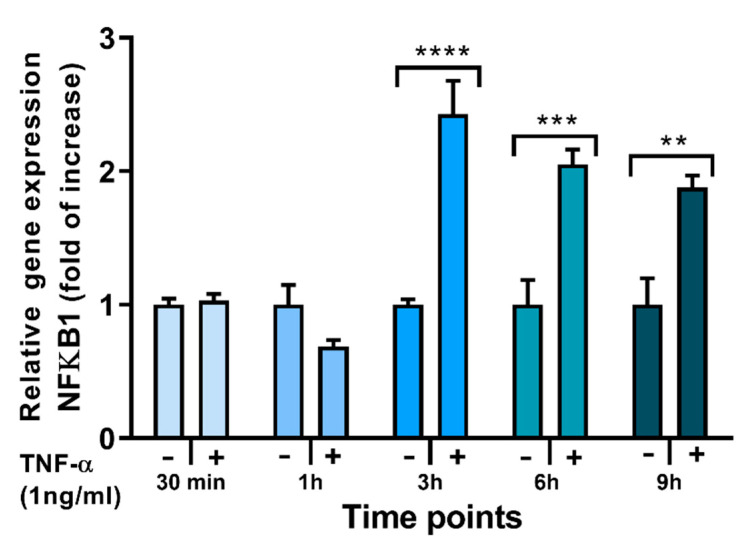
Expression of the NFκβ1 gene in EA.hy926 cells stimulated (+) with TNF-α (1 ng/mL) for different times up to 9 h or not stimulated (−). Cq values were normalised by the geometric mean of reference targets RPL13A and CYC1 genes. Bars are mean ± SEM of 3 samples from 1 experiment. Stimulated and unstimulated cells were compared at each time point by Student’s *t*-test. ** *p* < 0.01, *** *p* < 0.001, **** *p* < 0.001 for stimulated vs. unstimulated cells.

TNF-α stimulation is well known to activate the nuclear factor kappa-light-chain-enhancer of activated B cells (NFκB) signalling pathway, resulting in NFκB translocation to the nucleus and upregulation of genes encoding multiple inflammatory cytokines and chemokines including MCP-1, IL-6, IL-8, and RANTES as well as other inflammatory proteins including those encoding toll-like receptor (TLR)-4 and cyclooxygenase (COX)-2 (PTGS2). In addition, TNF-α induces expression of the NFκB1 gene as part of the process of regulating inflammation; hence, as well as expression of cytokine, chemokine, TLR-4 and COX-2 genes, expression of the NFκB1 gene can be used to monitor inflammation in response to TNF-α and to test the effects of potential pro- and anti-inflammatory interventions. Stimulation of ECs with TNF-α induced a time-dependent increase in NFκB1 mRNA (Figure 4).

**Figure 5:**
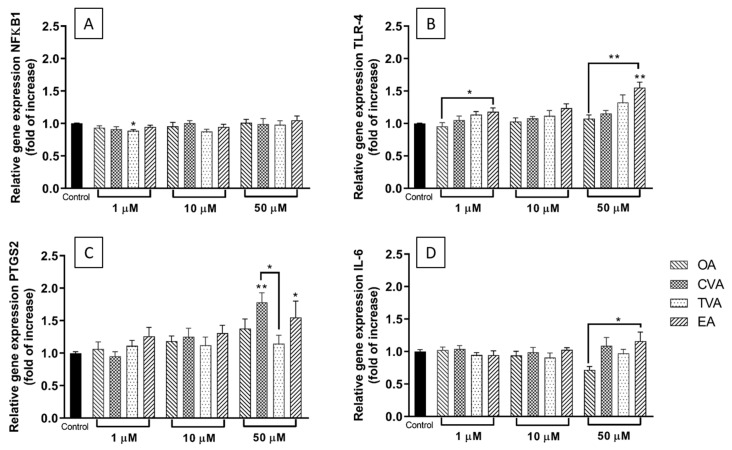
Expression of NFκβ1 (**A**), TLR-4 (**B**), PTGS2 (**C**), and IL-6 (**D**) genes in EA.hy926 cells preincubated for 48 h with 1, 10, and 50 µM of FA in DMEM containing 0.1% of ethanol (Control) followed by incubation with TNF-α (1 ng/mL) for 6 h. Cq values were normalised by the geometric mean of reference targets RPL13A and CYC1 genes. Bars are mean ± SEM of 9 samples performed in 3 experiments. Data were analysed using one-way ANOVA with Tukey’s as post hoc test. * *p* < 0.05, ** *p* < 0.01 between groups indicated by joined lines or vs. Control when there is no joined line. OA = oleic acid, CVA = *cis* vaccenic acid, TVA = *trans* vaccenic acid, EA = elaidic acid.

As shown in Figure 5A, preincubation with TVA at 1 µM reduced the relative expression of NFκB1 mRNA following TNF-α stimulation compared to what was seen with the TNF-α stimulated control cells (*p* < 0.05). The other FAs used did not induce any changes in NFκB1 relative expression.

Figure 5B shows that preincubation with EA at 1 µM increased TLR-4 gene expression following TNF-α stimulation compared to its *cis*-isomer OA (*p* < 0.05), while at 50 µM, the increase was significantly different from that with control or OA pretreated cells (both *p* < 0.01).

For the gene encoding cyclooxygenase COX-2 (PTGS2), only the highest FA concentration produced significant modulation: preincubation with either CVA or EA induced an increase in COX-2 gene expression following TNF-α stimulation compared to control (Figure 5C, *p* < 0.05 and *p* < 0.01, respectively). Additionally, the level was higher with CVA than with TVA (*p* < 0.05).

Preincubation with EA at a concentration of 10 µM increased TNF-α induced IL-6 gene expression compared to preincubation with OA (Figure 5D).

### 2.5. Effects of FAs on THP-1 Adhesion to EA.hy926 Cells

**Figure 6:**
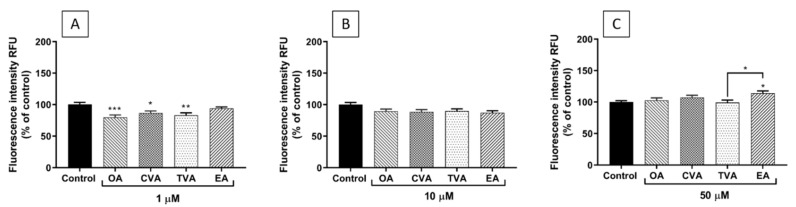
Adhesion of THP-1 cells (% of control) to EA.hy926 cells incubated for 48 h with DMEM containing 0.1% of ethanol (Control) or different concentrations (1 µM (**A**), 10 µM (**B**), 50 µM (**C**)) of FA, followed by incubation with TNF-α (1 ng/mL) for 24 h and then 1 h co-incubation with THP-1 cells. Bars are mean ± SEM of 9 samples performed in 3 experiments. Data were analysed using one-way ANOVA with Tukey post hoc test. * *p* < 0.05; ** *p* < 0.01; *** *p* < 0.001 between groups indicated by joined lines or vs. Control when there is no joined line. OA = oleic acid, CVA = *cis* vaccenic acid, TVA = *trans* vaccenic acid, EA = elaidic acid.

As shown in Figure 6A, preincubation of EA.hy926 cells with TVA, OA, or CVA at 1 µM for 48 h prior to TNF-α stimulation decreased the subsequent adhesion of THP-1 monocytes compared to control (*p* < 0.001, *p* < 0.05, and *p* < 0.01, respectively). When the FAs were used at 10 µM, none of them induced changes in monocyte adhesion compared to control (Figure 6B). Preincubation with EA at 50 µM increased monocyte adhesion compared to control (*p* < 0.05) and TVA pretreated cells (*p* < 0.05) (Figure 6C).

**Figure 7:**
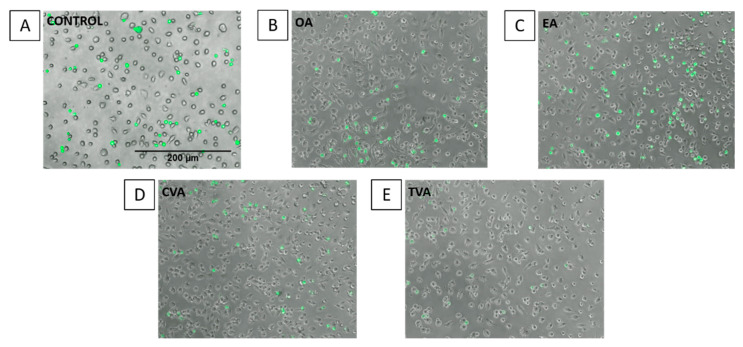
Images of THP-1 cell adhesion to EA.hy926 cells. Adhesion of THP-1 cells to EA.hy926 cells without preincubation with FA (control (**A**)) or with 48 h prior exposure to 50 µM oleic acid (**B**), elaidic acid (**C**), *cis* vaccenic acid (**D**), or *trans* vaccenic acid (**E**), followed by incubation with TNF-α (1 ng/mL) for 6 h and then 1 h co-incubation with calcein-labelled THP-1 cells. Attached THP-1 cells were visualised by fluorescence microscopy (Nikon Elipse Ti) at a magnification of 100 × under transmitted light.

Images taken under the fluorescence microscope agree with the quantitative results, showing a higher number of THP-1 monocytes (green spots) when ECs were preincubated with EA (Figure 7).

### 2.6. Effects of FAs on the Expression of ICAM-1 on the Surface of EA.hy926 Cells

**Figure 8:**
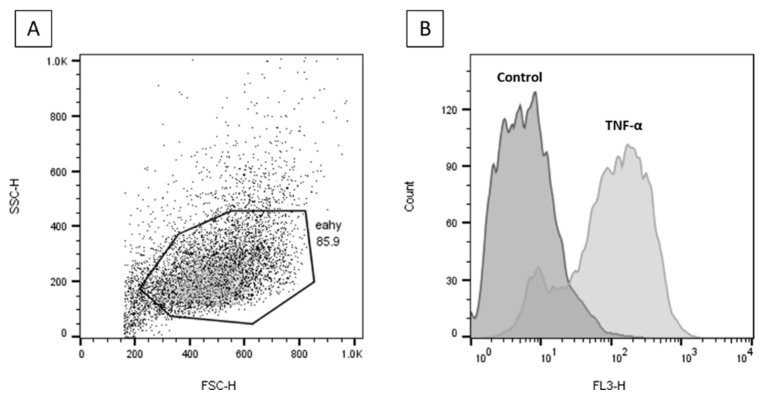
Flow cytometry plots for cell surface ICAM-1 analysis. (**A**) Gated unstained TNF-α stimulated EA.hy926 cells. (**B**) Gated unstimulated (Control) and TNF-α stimulated (PE-CyTM 5)-conjugated CD54 (ICAM-1) antibody stained EA.hy926 cells.

Incubation of EA.hy926 cells with TNF-α significantly upregulated cell surface ICAM-1 expression (Figure 8).

**Figure 9:**
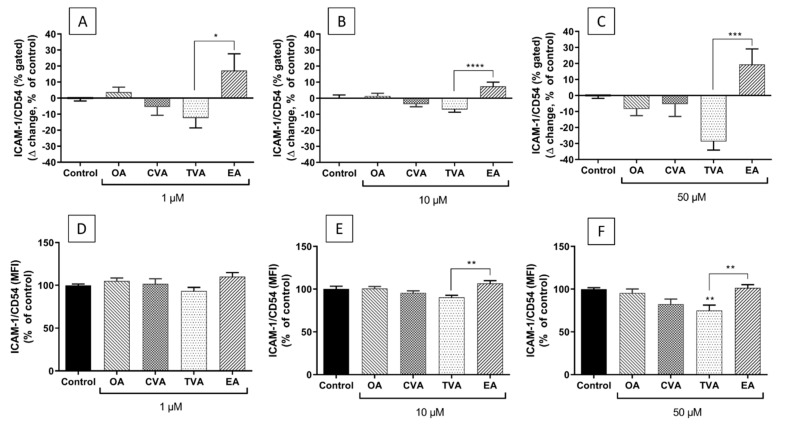
Cell surface expression of ICAM-1 as % of EA.hy926 cells gated (∆ change, % of control) (**A**–**C**) and as median fluorescence intensity (MFI, % of control) (**D**–**F**) after preincubation for 48 h with DMEM containing 0.1% ethanol (Control) or different concentrations (1 µM; 10 µM; 50 µM) of FA, followed by incubation with TNF-α (1 ng/mL) for 6 h. Bars are mean ± SEM of 9 samples performed in 3 experiments. Data were analysed using one-way ANOVA with Tukey’s post hoc test. * *p* < 0.05; ** *p* < 0.01; *** *p* < 0.001, **** *p* < 0.0001 between groups indicated by joined lines or vs. Control when there is no joined line. OA = oleic acid, CVA = *cis* vaccenic acid, TVA = *trans* vaccenic acid, EA = elaidic acid.

Preincubation with FAs prior to TNF-α stimulation was shown to have differential effects on surface ICAM-1 expression depending on the individual FA and on FA concentration. Preincubation with TVA showed a trend to reduce the % of cells expressing ICAM-1 with all the concentrations used, behaving significantly differently from EA (Figure 9A–C, *p* < 0.05, *p* < 0.0001, and *p* < 0.001, respectively).

The differences in the % of cells expressing ICAM-1 were consistent with the differences in the levels of ICAM-1 expression on the surface of positive cells (i.e., MFI). As shown in Figure 9D–F, preincubation with TVA tended to reduce the level of ICAM-1 expression on EA.hy926 cells subsequently stimulated with TNF-α, inducing a significant effect when used at 50 µM (*p* < 0.01). Preincubation with EA tended to induce the opposite effect, behaving significantly differently from TVA when used at 10 and 50 µM (both *p* < 0.01).

## 3. Discussion

With the purpose of understanding the possible differential effects and mechanisms through which TFAs could affect normal functioning of human endothelial tissues in relation to CVD development, the present study compared the effects of 18-carbon *cis* and *trans* FA isomers on the inflammatory response in cultured EA.hy926 ECs. These cells are derived from human umbilical vein ECs, which are perhaps the most widely studied type of EC. Their responses to inflammatory stimuli are similar to those seen with ECs from adults [23]. The effects of the TFAs were investigated using cells subsequently stimulated with TNF-α, indicating the ability of the TFAs to modulate the response to a classic inflammatory stimulus. In all cases, effects of TFAs were compared to cells incubated without additional FAs and to cells exposed to comparator 18-carbon *cis* FAs. Overall, this study showed differential effects of the FAs tested, mainly in terms of the effects of ruminant vs. industrial 18-carbon TFAs, and some differences between the *cis* and *trans* isomers.

In relation to the effects of the FAs on the production of inflammatory mediators by ECs, preincubation with EA increased or tended to increase the levels of most of the cytokines, chemokines, and adhesion molecules measured after inflammatory stimulation, while TVA showed a neutral effect. These results are consistent with the findings on gene expression. Here preincubation with EA induced a significantly enhanced upregulation of TLR-4 and COX-2 gene expression when used at 50 µM, which could be considered as pro-inflammatory. In contrast, preincubation with TVA induced a significant reduction in the NFκB1 gene expression when used at 1 µM and tended to do the same when used at 10 µM, which could be considered as anti-inflammatory.

When adhesion of monocytes (THP-1 cells) to the endothelial monolayer and the expression of ICAM-1 on the endothelial cell surface were assessed, differences between EA and TVA were also observed. Preincubation with EA induced a significant increase of THP-1 cell adhesion to ECs when used at 50 µM. EA showed a trend to increase both the % of gated ICAM-1 positive cells and the level of surface expression of ICAM-1 at all the concentrations used. Again, these effects could be regarded as pro-inflammatory and pro-atherogenic. In contrast, preincubation with TVA reduced THP-1 monocyte adhesion when used at 1 µM and tended to decrease both the % of gated ICAM-1 positive cells and the level of surface expression of ICAM-1 at all the concentrations used, reducing the latter significantly when used at 50 µM. These effects could be considered to be anti-atherogenic.

The current study suggests that EA and TVA have opposite effects on EC responses to inflammatory stimulation. Several studies have described that EA and other iTFAs have deleterious effects on health outcomes in humans: a high dietary intake or high blood/tissue levels were associated with CHD, systemic inflammation, endothelial dysfunction, and possibly inflammation in the central nervous system [15,24,25,26,27]. Accordingly, a recent study showed a positive association between plasma EA levels with long-term total mortality in a subset of NHANES participants (cycle 1999–2000) [28]. Animal models have shown that EA enhances inflammatory parameters in cerebrospinal fluid and blood, increases insulin resistance, alters lipid profiles, and causes hepatic damage [29,30]. In vitro studies have described that EA (100 μM) induces the expression of ICAM-1 and vascular cell adhesion molecule (VCAM)-1 on the surface of aortic ECs, increases the expression of ICAM-1 and VCAM-1 mRNA, and also leukocyte adhesion, phosphorylation of NFκB, and reactive oxygen species generation in these cells [18]. Experiments with another human EC model (microvascular ECs) showed that exposure to EA (100 μM) increased NFκB activation as measured by IL-6 levels and phosphorylation of IκBα, increased superoxide production, and impaired insulin signaling and nitric oxide production [17], suggesting pro-inflammatory actions of EA. In the current study, using a maximum concentration that is half of what other authors have used (50 μM), it was shown that EA exposure to EA.hy926 cells increased inflammatory mediator levels (MCP-1, RANTES, and IL-8) in response to TNF-α. Increased levels of these mediators are related to a pro-inflammatory state that could lead to the development of atherosclerosis and endothelial dysfunction. For example, MCP-1 is one of the key chemokines that regulate migration and infiltration of monocytes/macrophages into the subendothelial space, being considered an early indicator of endothelial dysfunction [31].

EA also showed a trend to enhance TNF-α-induced surface expression of ICAM-1. This adhesion molecule is involved in the leukocyte-endothelium interaction and the regulation of vascular permeability [32]. Because adhesion is one of the earliest steps of inflammation in atherosclerosis, followed by migration of blood leukocytes into the subendothelial space, the increase in adhesive properties of ECs may be an important mechanism by which dietary iTFAs exert their pro-inflammatory and pro-atherogenic effects [18]. In agreement with this, EA induced a significant increase of THP-1 cell adhesion to ECs.

The evidence about the health-related effects of rTFAs is not consistent, especially when comparing the results from animal models with outcomes in human studies. It has been shown that TVA can lower fasting triglycerides, total cholesterol, LDL cholesterol, and non-esterified FAs in animal models of dyslipidaemia [33,34,35]. Blewett et al. [36] also reported that short-term (3 wk) supplementation with TVA (1.5% *w/w*) normalised stimulated IL-2 and TNF-α production and increased IL-10 production in JCR-LA-cp rats, suggesting anti-inflammatory effects. In humans, the literature is inconsistent. While some authors have reported beneficial effects of rTFAs, others have shown opposite results. For example, Da Silva et al. [37] compared iTFA and rTFA in plasma phospholipids and their correlations with metabolic risk factors, including lipid profile, glycaemic profile, adiposity, and blood pressure, in a cohort composed of 100 healthy non-obese and 100 obese participants. They found that plasma rTFAs (TVA and *trans* palmitoleic acid) were associated with lower insulin levels and blood pressure and higher adiponectin levels, unlike their industrial counterpart (EA) which was associated with higher total cholesterol, triglycerides, and glycaemia [37]. On the other hand, Gebauer et al. [38] conducted a double-blind, randomised, crossover feeding trial in 106 healthy adults. They determined the effects of TVA, a conjugated linoleic acid (CLA9,11), and EA, in the context of highly controlled diets, on lipoprotein risk factors compared with a control diet. Their findings showed that both TVA and partially hydrogenated vegetable oil adversely affect atherogenic lipoproteins, with higher concentrations of LDL cholesterol, apoB, and triacylglycerol [38]. In contrast, studies in endothelial and other cell line models have reported beneficial effects of TVA [17,20,39,40], concordant to some extent with the results presented in the current study. Overall, this study showed that while the levels of the inflammatory mediators measured did not change with the exposure of the ECs to TVA, the cell surface expression of ICAM-1 decreased significantly at the highest TVA concentration used (50 μM).

Even though other studies have reported outcomes related to inflammation in relation to exposure to TVA, they are not the same outcomes as measured in the current research, which makes the comparison with other findings considerably harder in comparison to EA. For instance, a study on human microvascular ECs reported that TVA (100 μM) did not induce any inflammatory responses in comparison to EA and linoelaidic acid, specifically in relation to NFκB activation, levels of IL-6, and superoxide production [17]. Another study using human peripheral blood mononuclear cells showed that TVA decreased the percentage of both IL-2 and TNF-α expressing T-helper cells induced by alloreactive stimulation [39]. Krogager et al. [40] showed that TVA had no effect on proliferation of HepG2-SF cells or their metabolism of cholesterol in comparison to EA, assessed by proteome analysis. The study by Da Silva et al. [21] showed that TVA at concentrations above 25 μM significantly reduced the TNF-α induced expression of TNF-α, VCAM-1, and superoxide dismutase 2 genes in HUVECs. The same study reported that TVA induced a reduction in the gene expression of IL-8 and TNF in HepG2 cells [21]. Another recent study in HUVECs showed an increased protein expression of ICAM-1, VCAM-1, IL-6, and COX-2 and increased prostaglandin E_2_ secretion in cells exposed to TVA or EA (100 μM for 24 h) compared to control, although the inflammatory responses induced by EA were significantly higher than the ones observed with the TVA treatment. This study also reported that these TFAs induced reduced cell viability and cell membrane damage [41], in agreement with our observations for these two FAs at 100 μM.

In terms of the mechanism of action of these FAs, it seems plausible that TLR-4 and NFκB are involved. Preincubation with EA induced an increase in mRNA expression of TLR-4 and COX-2 in response to stimulation with TNF-α, but had no effect on NFκB1 mRNA expression. The TLR-4 pathway eventually activates NFκB signalling [42], which involves rapid increases in NFκB phosphorylation and nuclear translocation, and later (post 3 h stimulation) higher gene expression of NFκB genes. In the current study, EA did not affect NFκB1 gene expression 6 h post-TNF-α stimulation. We did not measure IκB or NFκB phosphorylation or NFκB translocation to the nucleus, so an effect of EA on the NFκB signalling pathway cannot be ruled out. Other authors have suggested that EA exerts pro-inflammatory actions in HUVECs through increased TLR-4 protein expression within lipid rafts [19]. The increased TLR-4 observed with preincubation with EA in the current study supports this, but we did not measure membrane levels of TLR-4 or TLR-4 associated with lipid rafts. Future investigations of the mechanism of action of EA in ECs should focus on these early signalling events. Although TVA had no effect at higher concentrations, at 1 μM it was able to reduce NFκB1 relative gene expression in TNF-α stimulated ECs.

The difference between the findings shown here and those of other studies can be related to the FA concentrations and cell line used and other experimental/methodological differences. In addition, other authors may not check the concentrations of the FAs used periodically or even at all. While conducting the current research, it was observed that TFAs can be unstable when stored, so not knowing precisely the FA concentration used in in vitro studies is not a negligible factor when analysing the effects of TFAs. In relation to the FA concentrations used in this study, a maximum of 50 μM was considered to avoid the toxic effects observed at 100 μM (indicated by lower cell viability at this concentration). It is important to consider that most of the studies in the literature use TFA concentrations of 100 μM average, reaching maximum values of 400 μM [43]. It is important to consider whether these concentrations are physiologically achievable and comparable to the amount of TFA that can be incorporated into human cells and tissues through the diet. There is limited information on this. Nevertheless, the highest TFAs levels reported in plasma in healthy adults correspond to 88 µM for EA and 74 µM for TVA [44].

In common with other FAs, TFAs are likely to exert most of their effects on inflammation following incorporation into the cell membrane [13]. From here they can potentially influence membrane fluidity, membrane protein function, lipid raft formation, and the generation of intracellular signals [13]. These effects may, in turn, affect transcription factors and gene expression [13]. It might be anticipated that the effects of FAs on inflammatory cell responses will be concentration dependent, because their incorporation into cells is concentration dependent, as demonstrated here for all four FAs investigated (Figure 2). As discussed already, EA had the most marked and consistent effects on ECs in the current study and these may be generally viewed as enhancing the response to the inflammatory stimulus TNF-α. EA had its greatest effects on MCP-1, IL-8, and RANTES production, on TLR-4 and PTGS2 gene expression, on THP-1 cell binding, and on ICAM-1 expression when used at a concentration of 50 µM. Although, some of these effects do appear to be concentration dependent, in some cases (ICAM-1, IL-8, and RANTES production) EA had a significant effect at 1 µM, but not at 10 µM. The reason for this is not immediately apparent but it may relate to the relatively small number of replicates and/or to inherent variations between the individual experiments including the precise incorporation of the FAs into the ECs. What is evident is that quite marked incorporation of the different FAs occurred when they were used at a concentration of 50 µM and it was at this concentration that the effects of EA (and TVA) were most marked. Thus, it seems likely that the effects of TFAs on inflammation are concentration dependent but that these effects may not become apparent until sufficient FA is incorporated into the cells.

In summary, the results of this study indicate that the ruminant-derived TVA has the potential to reduce some inflammatory responses of ECs related to atherosclerosis. In contrast, EA increased inflammatory responses of ECs to TNF-α stimulation. These findings suggest differential effects induced by the TFAs tested fitting with the idea that iTFAs and rTFAs can have different and perhaps opposing biological actions. The mechanisms through which these FAs influence the inflammatory response in ECs need further exploration, although TLR-4 and NFκB pathways are likely to be involved.

## 4. Materials and Methods

### 4.1. Endothelial Cell Model

EA.hy926 cells (ATCC, LGC standards, Middlesex, UK) were cultured in high glucose Dulbecco’s Modified Eagle Medium (DMEM) supplemented with 10% fetal bovine serum, 1% L-glutamine-penicillin-streptomycin solution, and 1% HAT (100 µM hypoxanthine, 0.4 µM aminopterin and 16 µM thymidine); medium and supplements were purchased from Sigma-Aldrich (Gillingham, UK). Cultures were maintained at 37 °C in humidified 95% air and 5% CO_2_. Before their use in experiments, cells were grown in T-175 flasks until confluent.

### 4.2. Fatty Acid (FA) Treatment

*Trans* vaccenic acid (TVA), elaidic acid (EA), *cis* vaccenic acid (CVA), and oleic acid (OA) (all from Cayman Chemicals, Cambridge, UK) were prepared as 1, 10, and 50 mM stock solutions in 100% ethanol. Before each experiment, the stock solutions were diluted in warm complete culture medium to yield final concentrations of 1, 10, and 50 μM. The corresponding control was a 0.1% ethanol solution diluted in complete medium. For the experiments, EA.hy926 cells were seeded in 96 well plates (for MTT assay and ELISA), 6 well plates (for RT-PCR, adhesion assay, flow cytometry), or T25 flasks (for gas chromatography), cultured in complete medium and exposed to different FAs for 48 h. Based on the conditions optimised for studying inflammatory responses of cultured EA.hy926 cells, after the FA exposure period, cells were incubated with TNF-α (1 ng/mL; 20 units/mL) for 6 or 24 h, depending on the assay to be performed.

### 4.3. MTT Assay for Cell Viability

Cell viability was assessed using the 3-(4,5-dimethylthiazol-2-yl)-2,5-diphenyltetrazolium bromide (MTT) assay which measures cellular mitochondrial activity. After the treatments, supernatant was removed and replaced with DMEM containing 0.05 mg/mL MTT (Sigma-Aldrich) (100 µL/well) and samples incubated at 37 °C for 4 h. Supernatants (75 µL) were removed and 75 µL of dimethylsulphoxide (Sigma-Aldrich) added. Absorbance was measured at 540 nm on a plate reader. The effects of FAs and TNF-α on cell viability were normalized to control (i.e., no FA or TNF-α, 0.1% ethanol) cultures (100%).

### 4.4. Gas Chromatography

The FA concentrations and the FA composition of EA.hy926 cells after culture with the FAs of interest were determined using gas chromatography. For FA concentration testing, each FA was diluted in full warm medium from the respective stock in 100% ethanol. For FA incorporation, cells were seeded in T25 flasks (5 × 10^5^ cells/mL) for 48 h with each FA at different concentrations. Afterwards, the cells were inspected under the microscope, scraped off, and counted with a Beckman Coulter cell counter. EA.hy926 cells were resuspended to have 1 × 10^6^ cells/800 µL of 0.9% NaCl solution.

Total lipid was extracted from cell pellets and culture medium, after adding an internal standard (C21:0), using chloroform/methanol (2:1 *v/v*) and NaCl (1 M). Lipid extracts were dried under nitrogen at 40 °C and then resuspended in toluene. FAs were released from the isolated lipids and simultaneously methylated by heating with 2% sulphuric acid in methanol at 50 °C for 2 h. The resulting FA methyl esters (FAMEs) were extracted into hexane and then separated and analysed by gas chromatography using conditions described by Fisk et al. [45]. FAME histograms produced were analysed with Agilent ChemStation software. Thirty-seven FAMEs were used as standard to identify FAs according to retention time and for software calibration. FAs are expressed as µg/10^6^ cells.

### 4.5. Multiplex Magnetic ELISA

Cell culture supernatants were assayed by Human Magnetic Luminex Screening Assay ELISA (R&D Systems, Minneapolis, MN, USA) to measure the concentration of inflammatory factors monocyte chemoattractant protein (MCP)-1, interleukin (IL)-6, IL-8, regulated upon activation, normal T cell expressed and presumably secreted (RANTES), and intercellular adhesion molecule (ICAM)-1. EA.hy926 cells were incubated with the FAs in 96 well plates (1 × 10^4^ cells/100 µL per well) for 48 h and then incubated with TNF-α for a further 24 h. Before the supernatants of each well were collected and stored at −80 °C until analysis, the cells were checked under the microscope. Assays were conducted in accordance with the instructions from the manufacturer. Plates were analysed on a calibrated Bio-Plex 200 analyser using Bio-Plex software (version 6.1, Bio-Rad Laboratories Inc., Berkeley, CA). Lower limits of detection (pg/mL) were IL-6, 1.7; IL-8, 1.8; MCP-1, 9.9; RANTES, 1.8; ICAM-1, 87.9. Due to differences in the ranges of fluorescence values among experiments, the results are presented as % of control.

### 4.6. RNA Isolation, cDNA Synthesis, and Real-Time PCR

Changes in relative gene expression were analysed by RT-PCR. EA.hy926 cells were incubated with FAs for 48 h followed by incubation with TNF-α (1 ng/mL) for 6 h. Taqman Gene Expression Primers (ThermoFisher Scientific, Waltham, MA, USA) were used to determine the expression of nuclear factor kappa B subunit 1 (NFκB1) (Hs00765730_m1), toll-like receptor (TLR)-4 (Hs00152939_m1), cyclooxygenase (COX)-2 (Hs00153133_m1), and IL-6 (HS00985639_m1). Total RNA was extracted from the cells using the ReliaPrep RNA cell Miniprep System (Promega, Southampton, UK). RNA quantity and quality were analysed by NanoDrop. Analysis of RNA using an Agilent Bioanalyzer (RNA Total Eukaryote 2100 Nano) was performed to determine RNA integrity through RIN scores. cDNA was synthesised from total RNA using GoScript Reverse Transcriptase (Promega). Housekeeping reference genes were determined using a geNorm Kit (Primerdesign, Camberley, UK). Quantification of relative gene expression was analysed using YWHAZ, (Hs01122445_g1), CYC1 (Hs00357717_m1), and RPL13A (Hs04194366_g1) as housekeeping genes.

### 4.7. THP-1 Monocyte Adhesion Assay

The adhesion of monocytes (THP-1 cells) to EA.hy926 cells was determined using the Vybrant Cell Adhesion Assay Kit (ThermoFisher Scientific). EA.hy926 cells were seeded in 96-well flat bottom plates (density of 2 × 10^5^ cells/mL, 1 × 10^5^ cell per well). After incubation with FAs for 48 h and then with TNF-α for 24 h, calcein-labelled THP-1 cells (5 × 10^4^ cells in 100 µL) were incubated with EA.hy926 cells for 1 h at 37 °C. Non-adherent THP-1 cells were removed by gentle washing, 100 µL PBS added to each well and co-cultures read on the Glomax Discover System (Promega). THP-1 monocyte adhesion was measured as a percentage of control (non-stimulated DMEM treated cells). Images of fluorescence-labelled THP-1 monocytes bound to EA.hy926 cells were taken with a Nikon Elipse Ti using NIS elements software (version 4.30).

### 4.8. Flow Cytometry

The expression of ICAM-1 (CD54) on the surface of EA.hy926 cells was determined through flow cytometry. EA.hy926 cells were seeded in six well plates (density of 6 × 10^5^ cells/mL). After incubation with FAs and then with TNF-α, the cells were detached, centrifuged, and stained with PE-Cy^TM^5-conjugated monoclonal anti-human CD54 (BD Biosciences, San Jose, CA) diluted in staining solution (2% bovine serum albumin in PBS) for 30 min at 4 °C in darkness. Mouse IgG1 κ (PE-Cy^TM^5) isotype was used as a negative control. After staining, cells were analysed by flow cytometry using a FACSCalibur flow cytometer (BD Biosciences). A total of 10,000 events were collected. Percentage of positive cells and median fluorescence intensity (MFI) were measured.

### 4.9. Data Analysis

Data are presented as mean ± SEM and were analysed by two-way analysis of variance (two-way ANOVA) or one-way ANOVA, followed by post hoc tests of pairwise differences. Analyses were performed using GraphPad Prism 6.0. Differences were considered significant when *p* < 0.05.

## Data Availability

Data can be made available by contacting the corresponding author.
